# The spread of cat-transmitted sporotrichosis due to *Sporothrix brasiliensis* in Brazil towards the Northeast region

**DOI:** 10.1371/journal.pntd.0009693

**Published:** 2021-08-30

**Authors:** Aurélio de Oliveira Bento, Alexandre Soares de Sena Costa, Soraia Lopes Lima, Manoella do Monte Alves, Analy Salles de Azevedo Melo, Anderson Messias Rodrigues, Walicyranison Plinio da Silva-Rocha, Eveline Pipolo Milan, Guilherme Maranhão Chaves

**Affiliations:** 1 Laboratório de Micologia Médica e Molecular, Departamento de Análises Clínicas e Toxicológicas, Universidade Federal do Rio Grande do Norte, Natal, Rio Grande do Norte, Brasil; 2 Laboratório Especial de Micologia, Disciplina de Infectologia, Universidade Federal de São Paulo, São Paulo, Brasil; 3 Departamento de Infectologia, Instituto de Medicina Tropical, Universidade Federal do Rio Grande do Norte, Natal, Rio Grande do Norte, Brasil; 4 Laboratório de Patógenos Fúngicos Emergentes, Divisão de Biologia Celular, Departamento de Microbiologia, Imunologia e Parasitologia, Escola Paulista de Medicina, Universidade Federal de São Paulo, São Paulo, Brasil; 5 Laboratório de Micologia Clínica, Departamento de Ciências Farmacêuticas, Universidade Federal da Paraíba, Brazil; Institute of Continuing Medical Education of Ioannina, GREECE

## Abstract

**Background:**

Sporotrichosis is a worldwide subcutaneous mycosis caused by *Sporothrix* spp. In the past, this infection was associated with armadillo hunting, horticulturists, miners, and gardeners, being considered an implantation mycosis acquired by plant debris injury. Nevertheless, since the late nineties, it has been considered a zoonotic disease in Brazil. Here we report a case series of 121 patients with cat-transmitted sporotrichosis seen in Northeast Brazil.

**Methodology/Principal findings:**

Patient’s demographic, clinical data, and length of treatment were recorded. In addition, a mycological examination and further PCR confirmation of species identification were performed. One hundred and twenty two patients were diagnosed with subcutaneous sporotrichosis from October 2016 to December 2019, while PCR revealed that 71 of them were due to *S*. *brasiliensis*. The majority of the individuals were female (n = 86; 70.5%). Patient’s age ranged from 5 to 87 years old. The clinical forms found were lymphocutaneous (58.2%) and fixed cutaneous (39.4%). Interestingly, 115 patients reported previous contact with cats diagnosed with sporotrichosis. Patients were successfully treated with itraconazole and potassium iodide.

**Conclusions/Significance:**

Our study adds important contributions for the investigation of the spread of cat-transmitted subcutaneous sporotrichosis in Brazil, specifically towards the Northeast region of a continental-size country. It will also help clinicians to be aware of the existence and importance to accurately diagnose sporotrichosis and treat patients with this infectious disease in the lowest income region of Brazil.

## Author summary

Sporotrichosis is an infectious disease caused by fungi belonging to *Sporothrix* species. These microorganisms can be found in nature, specifically in the soil and decomposing wood. In the past, sporotrichosis was known as a “rose gardener’s disease”. It is still commonly acquired through injuries with plant debris in most countries around the world. However, a remarkable host shift has happened, allowing *Sporothrix* to survive in feline hosts (domestic cats) in Brazil. Therefore, the novel discovered fungal species was named *Sporothrix brasiliensis*. This fungus can grow at a cat´s body temperature (approximately 39°C) and may be found on their claws, blood, and saliva. Because these animals usually bite and scratch each other and their owners, an alternative route of transmission emerged in Rio de Janeiro, Brazil, in the late nineties. Since then, the disease has become an epidemic, and here we demonstrated that it is spreading towards the Northeast region of the country (2,860 km away from Rio de Janeiro). One hundred and twenty one patients were diagnosed with cat-transmitted sporotrichosis, mainly women who work as cleaners or house cleaners and housewives. Nevertheless, all the patients were successfully treated mainly with an antifungal drug called itraconazole.

## Introduction

Sporotrichosis is the most frequent subcutaneous fungal infection that affects both genders without preference of race and age. It has a worldwide distribution, mainly in tropical and subtropical areas, including Africa, Asia, and South America [1]. This infection, which presents a subacute or chronic course, is described as an implantation mycosis by some authors [1,2]. Sporotrichosis has a traditional subcutaneous clinical presentation, known as ascending nodular lymphangitis, palpable gummy lesions that may ulcerate and fistulize, draining a purulent discharge [1–4]. Nevertheless, sporotrichosis may involve other body sites, including the eyes, muscles, fascia, pulmonary and meningeal sites, besides causing osteoarticular and disseminated diseases [5–7].

Classically the infection is established after traumatic inoculation into the skin with plant debris, thorns, and straw (sapronotic route) [8]. However, from the early 2000s, this infection has also been associated with animal scratches and bites, mainly felines [2]. In this case, sporotrichosis is considered a zoonotic infection due to the animal to human transmission [9], which is the main route responsible for outbreaks in endemic areas [10].

Sporotrichosis is historically associated with the thermally dimorphic fungus *Sporothrix schenckii* [11]. However, since 2007, Marimon and colleagues, based on phenotypic and genotypic analyses, proposed four new species: *S*. *globosa*, *S*. *brasiliensis*, *S*. *mexicana*, and *S*. *luriei*, formerly *S*. *schenckii* var. *luriei* [12]. These species (except *S*. *mexicana*) are currently usually reported as the “pathogenic clade”, whereas the remaining species are placed within an “environmental clade”, including less frequent agents of infections which present mid-to-low pathogenic potential to mammals, such as the *S*. *pallida* complex (*S*. *chilensis*, *S*. *mexicana*, *S*. *humicola*, and *S*. *pallida*) and the *S*. *stenoceras* complex [13–15].

In recent years the increased incidence of cat-transmitted sporotrichosis in Brazil has been largely described for quite a few states [9]. In Rio de Janeiro state, the infection is considered an urban epidemic, with more than 5,000 cases of diagnosed zoonotic sporotrichosis in the last 17 years [16]. Besides Rio de Janeiro, which may be considered the most heavily affected state, other studies conducted within different states such as Minas Gerais, Sao Paulo, Parana, Santa Catarina and Rio Grande do Sul have described cases of zoonotic sporotrichosis in the South and Southeast Brazil [17].

The presence of infected felines in densely populated urban areas precedes human infection, and it is also associated with the increased number of cases. As cat transmission to humans is the most common form of zoonotic sporotrichosis, veterinarians, technicians, and cat owners have been considered populations at risk of infection. In this scenario, zoonotic sporotrichosis is considered an important public health problem [9,18].

The epidemiology of cat-transmitted sporotrichosis is poorly investigated in the Northeast Brazil. There are only a few studies published in the literature describing feline sporotrichosis in Ceara, Pernambuco, Piaui, and Paraiba [13,18–21]. The epidemiological investigation of sporotrichosis in endemic areas is essential to report to the public entities about the necessity of politics of prevention and control of the infection [9]. Thus, this study aimed to describe the first cases of cat-transmitted human sporotrichosis of patients seen in the reference infectious disease hospital in Natal city, Rio Grande do Norte state, Northeast Brazil.

## Methods

### Ethics statement

All clinical and demographic data of the patient were collected in accordance with the Local Research Ethics Committee from the Liga Norte-Riograndense Contra o Cancer Hospital, approved under number 042/042/2012. The written consent was waived because of data anonymization and commitment to preserve the identity of the patients.

### Patients and sample collection

A prospective study was performed with 122 patients admitted at the Giselda Trigueiro Hospital, the infectious disease reference tertiary hospital in the Rio Grande do Norte state, Northeast Brazil, between October 2016 and December 2019. The patients were submitted to the clinical evaluation, and the type of lesions suggestive of sporotrichosis was recorded.

Clinical data were analyzed as follows: gender, age, origin, occupation, type of fungal exposure, duration, and type of lesion, body site of lesion, clinical presentation, established therapy, course of treatment, outcome of patient, and type of exposure.

The samples were collected by either punch biopsy of the skin or needle aspiration of the purulent material (inflammatory exudate) inside the nodules (both kept in saline solution for further processing). Mycological examinations were performed at the Medical and Molecular Mycology Laboratory, Clinical and Toxicological Analysis Department, Federal University of Rio Grande do Norte.

### Direct examination, culture, and microculture

Biopsy fragments were carefully cut into parallel slices sections, while secretions were centrifuged at 3500 rpm for 10 minutes. Mycological direct examination of the clinical sample was performed by clarification of samples with potassium hydroxide (KOH 20%) for 30 minutes. The samples were analyzed with optical microscopy (400x of magnification). Subsequently, 20 μL aliquots of the precipitate were inoculated at seven equidistant spots on the surface of Mycosel Agar (BD, NJ, USA). The Petri dish was incubated at 25°C and observed daily for up to a month. Transition to the yeast phase was performed with further colony subculture at 37°C for another 5–10 days in brain heart infusion broth (BHI; HIMEDIA, INDIA) [7].

The macromorphological characteristics of the colonies, such as diameter, surface aspect, and melanin production, were analyzed. The micromorphological aspects, including septation, hyphal presence or absence of pigment, and type of conidiogenesis, were observed for the filamentous phase. The presence of budding cigar-shaped yeast cells was observed for yeast colonies.

### Molecular identification

The isolates were cultured on Mycosel agar for seven days at 37°C, and DNA was extracted using The PrepMan Ultra Sample Preparation Reagent (Applied Biosystems), according to the manufacturer’s instructions. DNA concentration and purity were determined with a NanoDrop spectrophotometer (Thermo Fisher Scientific). *Sporothrix* sp. DNA was used for PCR reaction [22] using species-specific primers that targeted the calmodulin gene (*CAL*). Briefly, reactions were performed in a final volume of 25 μL, including 12.5 μL PCR Master Mix (Promega Corporation), consisting of 3 mM MgCl_2_, 400 mM each dNTPs, and 50 U/mL Taq Polymerase; 9.5 μL water, 1 μL each of forward and reverse primers (10 pmol/μL; Integrated DNA Technologies, USA), and 1 μL of target DNA [100 ng/μL]. The following primers were used: Sbra-F and Sbra-R, Ssch-F and Ssch-R, Sglo-Fand Sglo-R, Smex-F and Smex-R [22]. PCR products were size-separated by agarose gel electrophoresis, and the gel was stained in a 0.5 μg/mL ethidium bromide buffer solution (TAE) [22].

### Statistics and spatial analyses

Continuous variables were expressed as mean ± standard deviation (SD) and compared using Student´s t test. Categorical variables were expressed as frequencies and percentages and compared using Chi-square (X^2^), as appropriate. All tests were 2-tailed, where a *P*-value <0.05 and a confidence interval of 95% were determined to represent statistical significance. The Spearmen coefficient was used to asses a possible correlation between age and duration of treatment. Statistical analyses were performed using the JASP software, version 0.14.10. For construction of the spatial analysis maps, we used the QGIS software, version 3.20.1.

## Results

### Patients

A total of 122 patients diagnosed with subcutaneous sporotrichosis between October 2016 and December 2019 were included in the present study. Of the total, 86 corresponded to females (70.5%), representing the majority of cases. Patients’ age ranged from 5 to 87 years old with an average of 45 ± 19 years old, median of 43 years old, and mode of 34 years old. The lesions were mostly located on the upper limbs (n = 90; 73.8%), including the fingers, hand, arm and forearm; followed by the lower limbs (n = 15; 12.3%), such as foot, ankle, calf, and thigh; face, including eyelid lesions (n = 3; 2.5%), while 11.4% of them showed multiple lesions (due to multiple body sites injuries). The injury site of a single patient was not reported (Table 1).

**Table 1:** Demographic, clinical, mycological, and antifungal treatment data of 122 patients diagnosed with cat-transmitted subcutaneous sporotrichosis from October 2016 and December 2019 in Rio Grande do Norte, Northeast Brazil.

Patient ID	Sex/age	Occupation	Exposure	Mycological examination	PCR identification	Type of lesion	Clinical form	Treatment	Dosage	Treatment duration (weeks)
**Patient 1**	F/32	Cleaner	Scratching by cat	*Sporothrix* sp.	*Sporothrix brasiliensis*	Ulcer on left hand + left forearm lymph nodes	Lymphocutaneous	PI*	Sat sol**. 10 drops/d***	7
**Patient 2**	F/13	Student	Scratching by cat	*Sporothrix* sp.	*Sporothrix brasiliensis*	Ulcer on left eyelid + Lymph nodes	Lymphocutaneous	PI	Sat sol. 10 drops/d	7
**Patient 3**	F/35	Housewife	Contact with cat /No scratching	*Sporothrix* sp.	*Sporothrix brasiliensis*	Ulcers on right leg, both arms and thorax	Fixed + erythema nodosum	PI	Sat sol. 10 drops/d	13
**Patient 4**	M/28	IT Technician	Scratching by cat	Not performed	Not performed	Ulcer on abdomen and left forearm	Lymphocutaneous	ITR****	200mg/d (2 cap/d)	13
**Patient 5**	F/61	Housewife	Scratching by cat	*Sporothrix* sp.	*Sporothrix brasiliensis*	Ulcer in left hand	Lymphocutaneous	ITR	200mg/d (2 cap/d)	18
**Patient 6**	F/56	Housewife	Scratching by cat	*Sporothrix* sp.	*Sporothrix brasiliensis*	Ulcer on left hand	Lymphocutaneous	ITR	200mg/d (2 cap/d)	18
**Patient 7**	F/45	Housewife	Scratching by cat	Not performed	Not performed	Ulcer on right hand	Fixed	ITR	200mg/d (2 cap/d)	13
**Patient 8**	M/19	Student	Scratching by cat	*Sporothrix* sp.	*Sporothrix brasiliensis*	Ulcer on right arm	Fixed + erythema nodosum	ITR	200mg/d (2 cap/d)	13
**Patient 9**	F/31	Veterinary assistant	Scratching by cat	*Sporothrix* sp.	*Sporothrix brasiliensis*	Ulcer on right arm and handle	Fixed	ITR	200mg/d (2 cap/d)	13
**Patient 10**	M/47	Joiner	Scratching by cat	*Sporothrix* sp.	*Sporothrix brasiliensis*	Ulcer on left hand	Lymphocutaneous	ITR	200mg/d (2 cap/d)	13
**Patient 11**	F/71	Retail seller	Scratching by cat	Not performed	Not performed	Ulcer on right hand + lymphatic cord	Lymphocutaneous	ITR	200mg/d (2 cap/d)	13
**Patient 12**	M/51	Endemy investigator	Scratching by cat	*Sporothrix* sp.	*Sporothrix brasiliensis*	Ulcer on right hand	Fixed	ITR	200mg/d (2 cap/d)	13
**Patient 13**	F/23	Musician	Bite by cat	Not performed	Not performed	Ulcer on left hand	Lymphocutaneous	ITR	200mg/d (2 cap/d)	13
**Patient 14**	F/51	Phone operator	Scratching by cat	*Sporothrix* sp.	*Sporothrix brasiliensis*	Nodules on the back of left hand	Fixed	ITR	200mg/d (2 cap/d)	13
**Patient 15**	F/73	Retired	Scratching by cat	*Sporothrix* sp.	*Sporothrix brasiliensis*	Ulcerated plaque on the left hand. Nodules on left forearm.	Lymphocutaneous	ITR	200mg/d (2 cap/d)	13
**Patient 16**	M/43	Pet shop cleaner	Scratching by cat	*Sporothrix* sp.	*Sporothrix brasiliensis*	Ulcer on left hand	Lymphocutaneous	ITR	200mg/d (2 cap/d)	13
**Patient 17**	F/55	Seamstress	Infected cat / No contact	Not performed	Not performed	Nodules on left forearm and hand	Lymphocutaneous	ITR	200mg/d (2 cap/d)	Not informed
**Patient 18**	F/68	Retired	Not informed	Not performed	Not performed	Ulcer on right handle	Fixed	ITR	200mg/d (2 cap/d)	13
**Patient 19**	F/31	Receptionist	Scratching by cat	*Sporothrix* sp.	*Sporothrix brasiliensis*	Ulcerated nodule on left 5th chirodactyl + left axillary lymph node + arthralgias	Lymphocutaneous	ITR	200mg/d (2 cap/d)	13
**Patient 20**	F/45	Maid	Infected cat / No contact	*Sporothrix* sp.	*Sporothrix brasiliensis*	Conjunctivitis in the right eye + ulcerated lesions in the Upper Left Limb	Lymphocutaneous + ocular	ITR	200mg/d (2 cap/d)	13
**Patient 21**	F/47	Not informed	Infected cat / No contact	Not performed	Not performed	Nail bed ulcer + Nodules on left arm and forearm	Lymphocutaneous	ITR	200mg/d (2 cap/d)	13
**Patient 22**	F/44	Builder	Infected cat / No contact	*Sporothrix* sp.	*Sporothrix brasiliensis*	Ulcerated lesion on the 4th left chirodactyl + epitroclear adenomegaly	Lymphocutaneous/Membranous	ITR	200mg/d (2 cap/d)	13
**Patient 23**	F/55	Geologist	Infected cat/No contact	*Sporothrix* sp.	*Sporothrix brasiliensis*	Ulcerated Plaque on Left Foot	Fixed	ITR	200mg/d (2 cap/d)	13
**Patient 24**	F/85	Housewife	Scratching by cat	Not performed	Not performed	Ulcer on both hands	Fixed	ITR	200mg/d (2 cap/d)	12
**Patient 25**	M/71	Retired	Scratching by cat	Not performed	Not performed	Ulcero on right shoulder and handle	Lymphocutaneous	ITR	200mg/d (2 cap/d)	13
**Patient 26**	F/56	Housewife	Scratching by cat	Not performed	Not performed	Left calf ulcer + sweet syndrome	Fixed	ITR	200mg/d (2 cap/d)	13
**Patient 27**	M/78	Retired	Scratching by cat	Not performed	Not performed	Ulcer-crusted nodule	Lymphocutaneous	ITR	200mg/d (2 cap/d)	13
**Patient 28**	F/17	Housewife	Infected cat / No contact	Not performed	Not performed	Fistulized nodules on left and right lower limb	Lymphocutaneous	ITR	200mg/d (2 cap/d)	9
**Patient 29**	F/35	Housewife	Infected cat / Contact	Not performed	Not performed	Ulcers on left calf	Fixed	ITR	200mg/d (2 cap/d)	8
**Patient 30**	F/40	Retail seller	Infected cat / No contact	*Sporothrix* sp.	*Sporothrix brasiliensis*	Ulcer on left handle	Fixed	ITR	200mg/d (2 cap/d)	13
**Patient 31**	F/34	Beautician	Scratching by cat	Not performed	Not performed	Ulcers on 2nd and 3rd left chirodactyl	Fixed	ITR	200mg/d (2 cap/d)	13
**Patient 32**	F/33	Not informed	Scratching by cat	*Sporothrix* sp.	*Sporothrix brasiliensis*	Ulcers nodules on left fingers	Lymphocutaneous	ITR	200mg/d (2 cap/d)	13
**Patient 33**	F/78	Housewife	Scratching by cat	*Sporothrix* sp.	*Sporothrix brasiliensis*	Ulcer on left hand + adenomegaly	Lymphocutaneous	ITR	200mg/d (2 cap/d)	9
**Patient 34**	M/50	Pet Shop Assistant	Scratching by cat	Not performed	Not performed	Ulcer on left handle + multiform erythema	Lymphocutaneous + erythema multiforme	ITR	200mg/d (2 cap/d)	13
**Patient 35**	F/85	Housewife	Infected cat / No contact	*Sporothrix* sp.	*Sporothrix brasiliensis*	Ulcer on 3rd chirodactyl of the left hand	Lymphocutaneous	ITR	200mg/d (2 cap/d)	13
**Patient 36**	F/63	Retired	Bite by cat	Not performed	Not performed	Ulcers on left forearm + lymphatic cord	Lymphocutaneous	ITR	200mg/d (2 cap/d)	13
**Patient 37**	M/24	IT	Infected cat / No contact	*Sporothrix* sp.	*Sporothrix brasiliensis*	Ulcered lesion on left handle	Fixed	ITR	200mg/d (2 cap/d)	19
**Patient 38**	M/55	Teacher	Bite by cat	*Sporothrix* sp.	*Sporothrix brasiliensis*	Nodules on right hand and handle + lymphangitis	Lymphocutaneous	ITR	400mg/d (4 cap/d)	13
**Patient 39**	F/42	Maid	Scratching by cat	*Sporothrix* sp.	*Sporothrix brasiliensis*	Violaceous nodule on right thumb + central ulceration	Fixed	ITR	200mg/d (2 cap/d)	13
**Patient 40**	F/54	Retired	Scratching by cat	Not performed	Not performed	Nodules on left hand, forearm, arm and collarbone	Lymphocutaneous	ITR	200mg/d (2 cap/d)	11
**Patient 41**	F/45	Nursing technician	Contact with cat / No injury	*Sporothrix* sp.	*Sporothrix brasiliensis*	Nodules on the back of the left hand	Lymphocutaneous	ITR	200mg/d (2 cap/d)	16
**Patient 42**	F/63	Cat rescuer	Infected cat / No contact	*Sporothrix* sp.	*Sporothrix brasiliensis*	Ulcer on left arm + lymphatic cord	Lymphocutaneous	ITR	200mg/d (2 cap/d)	13
**Patient 43**	F/32	Kitchen assistant	Bite by cat	*Sporothrix* sp.	*Sporothrix brasiliensis*	Ulcer on the back of the left hand + perilesional nodule + lymphangitis up to elbow + epitroclear adenopathy + conjunctival infiltrate	Lymphocutaneous + Hypersensitivity	ITR + PRE	200mg/d (2 cap/d)	12
**Patient 44**	F/41	Health agent	Scratching by cat	*Sporothrix* sp.	*Sporothrix brasiliensis*	Ulcer + ganglia + lymphatic cord on left leg	Lymphocutaneous	ITR	200mg/d (2 cap/d)	13
**Patient 45**	F/72	Retired	Contact with cat	Not performed	Not performed	Ulcerated plate on right hand + erythema nodosum	Fixed + erythema nodosum	ITR	200mg/d (2 cap/d)	13
**Patient 46**	F/70	Not informed	Scratching by cat	*Sporothrix* sp.	*Sporothrix brasiliensis*	Erythematosus plaque + central ulcer on right calf + violacea erythematosus + plates on digital pulps and palms	Fixed + erythema multiforme	ITR + PRE	200mg/d (2 cap/d)	15
**Patient 47**	F/43	Teacher	Bite by cat	*Sporothrix* sp.	*Sporothrix brasiliensis*	Ulcer on right calf, right and left arm + arthralgias	Lymphocutaneous	ITR	200mg/d (2 cap/d)	13
**Patient 48**	F/54	Not informed	Not informed	Not performed	Not performed	Ulcer on left leg and arm + erythema multiforme	Fixed + erythema multiforme	ITR	200mg/d (2 cap/d)	12
**Patient 49**	F/21	Student	Scratching and bite by cat	*Sporothrix* sp.	*Sporothrix brasiliensis*	Ulcer on left forearm	Lymphocutaneous	ITR	200mg/d (2 cap/d)	13
**Patient 50**	F/61	Maid	Cat without injury	Not performed	Not performed	Ulcers on left calf	Fixed	ITR	200mg/d (2 cap/d)	8
**Patient 51**	F/64	Maid	Bite by cat	*Sporothrix* sp.	*Sporothrix brasiliensis*	Ulcerated and nodular lesions in left forearm + satellites erythematous and papulous lesions + nodules in the left arm	Lymphocutaneous	ITR	200mg/d (2 cap/d)	13
**Patient 52**	F/22	Student	Contact with cat	Not performed	Not performed	Ulcer on the 3rd left chirodactyl + ganglia on left arm and forearm	Lymphocutaneous	ITR	200mg/d (2 cap/d)	13
**Patient 53**	F/32	Nursing technician	Scratching and bite by cat	*Sporothrix* sp.	*Sporothrix brasiliensis*	Ulcer on left hand and fist +modules in left arm	Lymphocutaneous	ITR	200mg/d (2 cap/d)	13
**Patient 54**	M/41	Doorman	Scratching by cat	*Sporothrix* sp.	*Sporothrix brasiliensis*	Ulcer on the 4th chirodactyl + nodule on the right arm	Lymphocutaneous	ITR	200mg/d (2 cap/d)	13
**Patient 55**	F/50	Maid	Scratching by cat	Not performed	Not performed	Ulcer on left hand + injuries on knees and right elbow	Fixed + Hypersensitivity	ITR	200mg/d (2 cap/d)	16
**Patient 56**	M/58	Retired	Scratching by cat	*Sporothrix* sp.	*Sporothrix brasiliensis*	Multiple ulcers on the left hand and forearm + sporotrichotic rosary	Lymphocutaneous	ITR	200mg/d (2 cap/d)	25
**Patient 57**	F/52	Housewife	Bite by cat	Not performed	Not performed	No ulcer + erythema nodosum + lymphatic cord on left forearm	Lymphocutaneous + erythema nodosum	ITR	200mg/d (2 cap/d)	15
**Patient 58**	M/14	Student	Bite by cat	Not performed	Not performed	Crusted plaque	Fixed	ITR	200mg/d (2 cap/d)	Not infomrmed
**Patient 59**	F/17	Student	Bite by cat	Not performed	Not performed	Left upper Limb Injury + lymphatic cord	Lymphocutaneous	ITR	200mg/d (2 cap/d)	4
**Patient 60**	F/42	Not informed	Bite by cat	Not performed	Not performed	Lesion on distal interphalangeal joint of the 4th left chirodactyl	Fixed	ITR	200mg/d (2 cap/d)	13
**Patient 61**	F/53	Farmer	Scratching and bite by cat	Not performed	Not performed	Disseminated polymorph erythema	Erythema multiforme	ITR + PRE		8
**Patient 62**	F/70	Housewife	Scratching by cat	*Sporothrix* sp.	*Sporothrix brasiliensis*	Multiples	Lymphocutaneous	ITR	200mg/d (2 cap/d)	Not informed
**Patient 63**	M/30	Maid	Scratching by cat	*Sporothrix* sp.	*Sporothrix brasiliensis*	Ulcers in the upper limb + lymphatic cord	Lymphocutaneous	ITR	200mg/d (2 cap/d)	17
**Patient 64**	F/45	Nursing technician	Scratching by cat	Not performed	Not performed	Ulcer on the 2nd right chirodactyl + lymphatic cord on right upper limb	Lymphocutaneous	ITR	200mg/d (2 cap/d)	11
**Patient 65**	F/21	Not informed	Bite by cat	Not performed	Not performed	Ulcer in the 1st right and left chirodactyl	Fixed	ITR	200mg/d (2 cap/d)	13
**Patient 66**	F/29	Tax auditor	Scratching by cat	Not performed	Not performed	Ulcer below the right clavicle	Fixed	ITR	200mg/d (2 cap/d)	11
**Patient 67**	F/77	Housewife	Scratching by cat	Not performed	Not performed	Ulcer on the distal 1/3 of the left forearm	Fixed	ITR	200mg/d (2 cap/d)	10
**Patient 68**	F/77	Housewife	Scratching by cat	Not performed	Not performed	Ulcer on the 1st right chirodactyl + lymphatic cord on right upper limb	Lymphocutaneous	ITR	200mg/d (2 cap/d)	13
**Patient 69**	M/18	Not informed	Scratching and bite by cat	Not performed	Not performed	Ulcers and nodules on right upper limb	Lymphocutaneous	ITR	200mg/d (2 cap/d)	13
**Patient 70**	F/43	Housewife	Scratching by cat	Not performed	Not performed	Disseminated nodular lesions—spontaneous regression	Lymphocutaneous	ITR	200mg/d (2 cap/d)	1
**Patient 71**	F/30	Not informed	Scratching by cat	Not performed	Not performed	Lesions on lower limbs spreading to the trunk, inguinal region, cervical, face, and abdomen	Lymphocutaneous	ITR	200mg/d (2 cap/d)	8
**Patient 72**	F/34	Teacher	Scratching by cat	Not performed	Not performed	Ulcer on left knee	Fixed	ITR	200mg/d (2 cap/d)	13
**Patient 73**	F/18	Student	Scratching by cat	*Sporothrix* sp.	*Sporothrix brasiliensis*	Nodule on left thigh	Fixed	ITR	200mg/d (2 cap/d)	13
**Patient 74**	F/37	Veterinary Doctor	Scratching by cat	Not performed	Not performed	Ulcer on left thumb + lymphatic cord on left upper limb	Lymphocutaneous	ITR	200mg/d (2 cap/d)	13
**Patient 75**	F/40	Artisan	Indirect contact with cat	Not performed	Not performed	Scar injury on 2nd right chirodacty	Fixed	ITR	200mg/d (2 cap/d)	6
**Patient 76**	M/41	Businessman	Scratching by cat	Not performed	Not performed	Erythematous lesions on right upper limb + Lymphnodemegaly	Lymphocutaneous	ITR	200mg/d (2 cap/d)	13
**Patient 77**	F/87	Housewife	Scratching by cat	Not performed	Not performed	Ulcer on left thumb + sporotrichotic rosary on forearm	Lymphocutaneous	ITR	200mg/d (2 cap/d)	8
**Patient 78**	M/34	Veterinary assistant	Scratching by cat	*Sporothrix* sp.	*Sporothrix brasiliensis*	Nodule on right handle	Fixed	ITR	200mg/d (2 cap/d)	4
**Patient 79**	F/43	Businessman	Bite by cat	*Sporothrix* sp.	*Sporothrix brasiliensis*	Ulcer on left thumb + nodular lesions on upper limbs, anterior thorax, interscapular region, face, and posterior legs	Fixed + Erythema multiforme	ITR	200mg/d (2 cap/d)	8
**Patient 80**	F/73	Housewife	Scratching and bite by cat	Not performed	Not performed	Lesion on right heel	Fixed	ITR	200mg/d (2 cap/d)	15
**Patient 81**	M/24	Bricklayer	Scratching by cat	Not performed	Not performed	Lesion on right ankle	Fixed	ITR	200mg/d (2 cap/d)	8
**Patient 82**	M/37	Policeman	Scratching by cat	Not performed	Not performed	Lesion on 2nd right chirodactyl	Lymphocutaneous	ITR	200mg/d (2 cap/d)	4
**Patient 83**	F/59	Administrative assistant	Cat without injury	*Sporothrix* sp.	*Sporothrix brasiliensis*	Lesion on left forearm	Fixed + Erythema multiforme	ITR	200mg/d (2 cap/d)	5
**Patient 84**	F/34	Unemployed	Not informed	Not performed	Not performed	Scar on right thigh	Fixed	ITR	200mg/d (2 cap/d)	Not informed
**Patient 85**	F/55	Housewife	Scratching by cat	Not performed	Not performed	Erythematous papules on 5th right chirodactyl	Lymphocutaneous	ITR	200mg/d (2 cap/d)	16
**Patient 86**	M/23	Bricklayer	Scratching by cat	Not performed	Not performed	Ulcer on left hand	Lymphocutaneous	ITR	200mg/d (2 cap/d)	6
**Patient 87**	M/34	Psychologist	Scratching and bite by cat	Not performed	Not performed	Ulcer on right leg	Fixed	ITR	200mg/d (2 cap/d)	4
**Patient 88**	M/50	Driver	Scratching and bite by cat	Not performed	Not performed	Ulcer on 2nd right chirodactyl + nodule on right upper limb	Lymphocutaneous	ITR	200mg/d (2 cap/d)	17
**Patient 89**	F/47	Housewife	Bite by cat	Not performed	Not performed	Ulcer on right upper limb + lymphatic cord	Lymphocutaneous + erythema multiforme	ITR	200mg/d (2 cap/d)	13
**Patient 90**	F/37	Retail seller	Blood of cat	*Sporothrix* sp.	*Sporothrix brasiliensis*	Ulcer on right maleolo + nodule on thigh	Lymphocutaneous	ITR	200mg/d (2 cap/d)	8
**Patient 91**	F/66	Retail seller	Bite by cat	Not performed	Not performed	Edema + Erythema on left thumb	Fixed	ITR	200mg/d (2 cap/d)	Not informed
**Patient 92**	M/33	Retail seller r	Scratching by cat	*Sporothrix* sp.	*Sporothrix brasiliensis*	Sporotrichotic rosary on right upper limb + ulcer on right handle	Lymphocutaneous	ITR	200mg/d (2 cap/d)	10
**Patient 93**	M/9	Student	Scratching by cat	Not performed	Not performed	Lesion on left forearm	Fixed	ITR	200mg/d (2 cap/d)	9
**Patient 94**	F/41	Housewife	Bite by cat	Not performed	Not performed	Ulcerous lesion on upper limb (3rd left chirodactyl) + lymphatic cord	Lymphocutaneous	ITR	200mg/d (2 cap/d)	11
**Patient 95**	F/62	Seller	Bite by cat	Not performed	Not performed	Ulcer on left arm + nodule on right arm	Fixed	ITR	200mg/d (2 cap/d)	9
**Patient 96**	F/47	Teacher	Scratching and bite by cat	Not performed	Not performed	Ulcer on right hand + nodule on right upper limb + polymorphic erythema	Lymphocutaneous + erythema multiforme	ITR	200mg/d (2 cap/d)	8
**Patient 97**	F/73	Housewife	Scratching by cat	Not performed	Not performed	Ulcer on left handle + nodule on left upper limb	Lymphocutaneous	ITR	200mg/d (2 cap/d)	8
**Patient 98**	F/20	Student	Scratching by cat	Not performed	Not performed	Ulcer on right handle + nodule on left upper limb + nodule on left forearm	Lymphocutaneous + erythema multiforme	ITR	200mg/d (2 cap/d)	17
**Patient 99**	F/69	Nursing technician	Bite by cat	*Sporothrix* sp.	*Sporothrix brasiliensis*	Ulcer on right thumb + lymphatic cord on right foot and leg	Lymphocutaneous	ITR	200mg/d (2 cap/d)	11
**Patient 100**	M/66	Retail seller	Scratching by cat	Not performed	Not performed	Ulcer on 4th right chirodactyl + epitroclear nodule + polymorphic erythema on hands	Fixed + erythema multiforme	ITR	200mg/d (2 cap/d)	11
**Patient 101**	F/26	Housewife	Scratching and bite by cat	*Sporothrix* sp.	*Sporothrix brasiliensis*	Ulcers on the left and right forearms + lymphatic cord + right epitroclear and axilar ganglia	Lymphocutaneous + erythema nodosum	ITR	300mg/d (3 cap/d)	11
**Patient 102**	M/57	Photographer	Scratching by cat	*Sporothrix* sp.	*Sporothrix brasiliensis*	Ulcer on abdome	Fixed	ITR	200mg/d (2 cap/d)	9
**Patient 103**	F/5	Student	Scratching by cat	*Sporothrix* sp.	*Sporothrix brasiliensis*	Ulcer on right foreamr + lymphatic cord on left and right foreamrs	Lymphocutaneous	ITR	200mg/d (2 cap/d)	17
**Patient 104**	F/14	Student	Indirect contact with cat	Not performed	Not performed	Nodule on the left eyelid + left hemiface	Lymphocutaneous	ITR	200mg/d (2 cap/d)	18
**Patient 105**	F/50	Housewife	Scratching by cat	Not performed	Not performed	Nodule on left forearm + lymphatic cord until underarm	Lymphocutaneous	ITR	200mg/d (2 cap/d)	Not informed
**Patient 106**	M/57	Businessman	Indirect contact with cat	*Sporothrix* sp.	*Sporothrix brasiliensis*	Ulcer on left foreamr+ lymphatic cord until underarm	Lymphocutaneous	ITR	200mg/d (2 cap/d)	11
**Patient 107**	F/51	Seller	Scratching by cat	Not performed	Not performed	Ulcer on 2nd left and right chirodactyls + lymphatic cord on left arm	Lymphocutaneous	ITR	200mg/d (2 cap/d)	Not informed
**Patient 108**	F/40	Maid	Scratching by cat	*Sporothrix* sp.	*Sporothrix brasiliensis*	Ulcer on right palm	Fixed	ITR	300mg/d (3 cap/d)	9
**Patient 109**	F/87	Housewife	Scratching by cat	*Sporothrix* sp.	*Sporothrix brasiliensis*	Ulcer on left tibia	Fixed	ITR	200mg/d (2 cap/d)	11
**Patient 110**	M/64	Driver	Scratching by cat	Not performed	Not performed	Ulcer on 1st right chirodactyl + lymphatic cord on right upper limb	Lymphocutaneous	ITR	200mg/d (2 cap/d)	6
**Patient 111**	F/61	Housewife	Contact with cat	Not performed	Not performed	Ulcer on 1st right chirodactyl + lymphatic cord on right foreamr + Ulcer on 1st left chirodactyl + Breast cancer	Lymphocutaneous	ITR	300mg/d (3 cap/d)	17
**Patient 112**	M/32	Mechanic	Contact with cat	Not performed	Not performed	Ulcer on 5th right chirodactyl + lymphatic cord	Lymphocutaneous + erythema nodosum	ITR	200mg/d (2 cap/d)	Not informed
**Patient 113**	M/41	Bricklayer	Contact with cat	*Sporothrix* sp.	*Sporothrix brasiliensis*	crusted plaque lesion	Fixed	ITR	200mg/d (2 cap/d)	11
**Patient 114**	M/50	Gardener	Wire injury	*Sporothrix* sp.	*Sporothrix* sp.	Ulcerous crusted lesion on right back of hand	Fixed	ITR	300mg/d (3 cap/d)	7
**Patient 115**	M/61	Retired	Scratching by cat	Not performed	Not performed	Ulcer on back of left hand + lymphatic cord on left upper limb	Lymphocutaneous	ITR	200mg/d (2 cap/d)	11
**Patient 116**	F/44	Seller	Contact with cat / No scratching	Not performed	Not performed	Ulcer on 5th right chirodactyl + lymphatic cord on right upper limb	Lymphocutaneous	ITR	200mg/d (2 cap/d)	Not informed
**Patient 117**	F/37	Bartender	Scratching by cat	Not performed	Not performed	Nodule on 2nd right chirodactyl + nodule on back of left hand + nodule on face	Fixed	ITR	200mg/d (2 cap/d)	9
**Patient 118**	M/33	Driver	Rose thorns	Not performed	Not performed	Ulcer on 1st left and right chirodactyl + lymphatic cord on left forearm and arm	Lymphocutaneous	ITR	200mg/d (2 cap/d)	14
**Patient 119**	M/13	Student	Contact with cat / No injury	Not performed	Not performed	Ulcerous nodule on jaw	Fixed	ITR	200mg/d (2 cap/d)	7
**Patient 120**	M/42	Public service worker	Bite by cat	Not performed	Not performed	Desquamative plaque with ulcer + lymphatic cord on back of the left hand	Lymphocutaneous	ITR	200mg/d (2 cap/d)	11
**Patient 121**	M/24	Engineer	Contact with cat / No injury	Not performed	Not performed	Ulcerous nodule on right arm	Fixed	ITR	200mg/d (2 cap/d)	7
**Patient 122**	F/16	Student	Scratching by cat	Not performed	Not performed	Nodules + Ulcer on left upper limb	Fixed	ITR	200mg/d (2 cap/d)	8

*Potassium iodide, *Saturated solution, ***Daily

**Fig 1:**
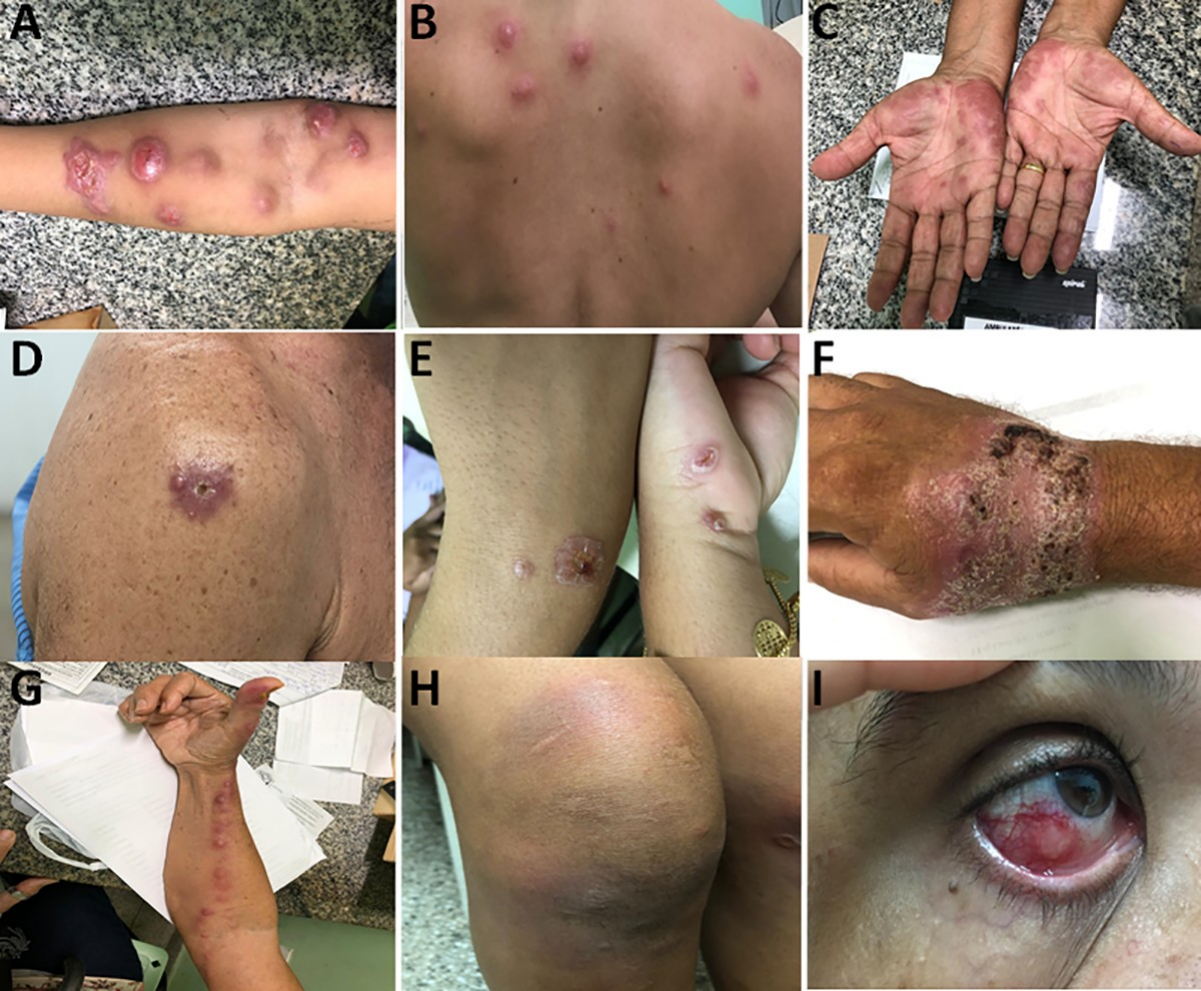
Clinical forms of cat-transmitted subcutaneous sporotrichosis of patients seen in Rio Grande do Norte state, Northeast Brazil. A = lymphocutaneous form. B = erythema nodosum. C = Polymorphic erythema. D = Fixed cutaneous form. E = Ulcer-crusted lesion. F = Crusted-plaque lesion. G = Sporotrichotic rosary. H = Reactive arthritis (arthralgia). I = Conjunctival infiltrate.

Regarding the clinical forms of sporotrichosis, 71 patients (58.2%) presented the lymphocutaneous form, three of them associated with erythema nodosum, four associated with polymorphic erythema, and two associated with ocular manifestation; while 48 (39.4%) had a fixed cutaneous form, with three of them presenting nodular erythema, while five patients showed polymorphic erythema. Besides these patients, patient 47 had a lymphocutaneous form, resulting from multiple cat bites on each mentioned body site: right and left arm and right calf. Patient 61 only presented polymorphic erythema, but no sporotrichoid lesions; patient 84 only had a scar on her left thigh, meaning that the lesion had started to heal after previous treatment (fixed cutaneous form). Altogether, they compose a population of 122 individuals (Table 1 and Fig 1).

In addition, three patients had a hypersensitivity reaction related to the lesion site. Atypical lesions were also observed in 4 patients, where two of them had an ulcer-crusted lesion (27 and 114) while the other two had a lesion in the form of a crusted plaque (58 and 113); and two patients (56 and 92) showed the sign of the sporotrichotic rosary. In addition, patients 19 and 47 had arthralgias, and patients 20 and 43 showed conjunctival infiltrate (Table 1 and Fig 1). No statistically significant correlation was observed for either the clinical form versus sex or clinical form versus age of patients (*P*˃0.05).

### Epidemiological data

The patients had different occupations, but 31 female patients were involved with domestic activities. Of the 122 patients, 115 (94.3%) reported previous contact with cats, either at work or at home. Among the total, 25 patients (21.7%) did not report any type of injury; 63 (54.8%) reported scratching by a cat; 18 (15.7%) reported bite, and nine cases (7.8%) reported both bite and scratch. In addition, one patient reported a glass injury (with remaining cat blood) that had previously cut an infected cat (diagnosed with sporotrichosis), and a single patient reported a barbed-wire fence injury. The time between the exposure and the appearance of the first sporotrichosis lesions lasted an average of 22 ± 18 days, with a median of 15 days and a 14-day mode, ranging from 2 to 90 days.

**Fig 2:**
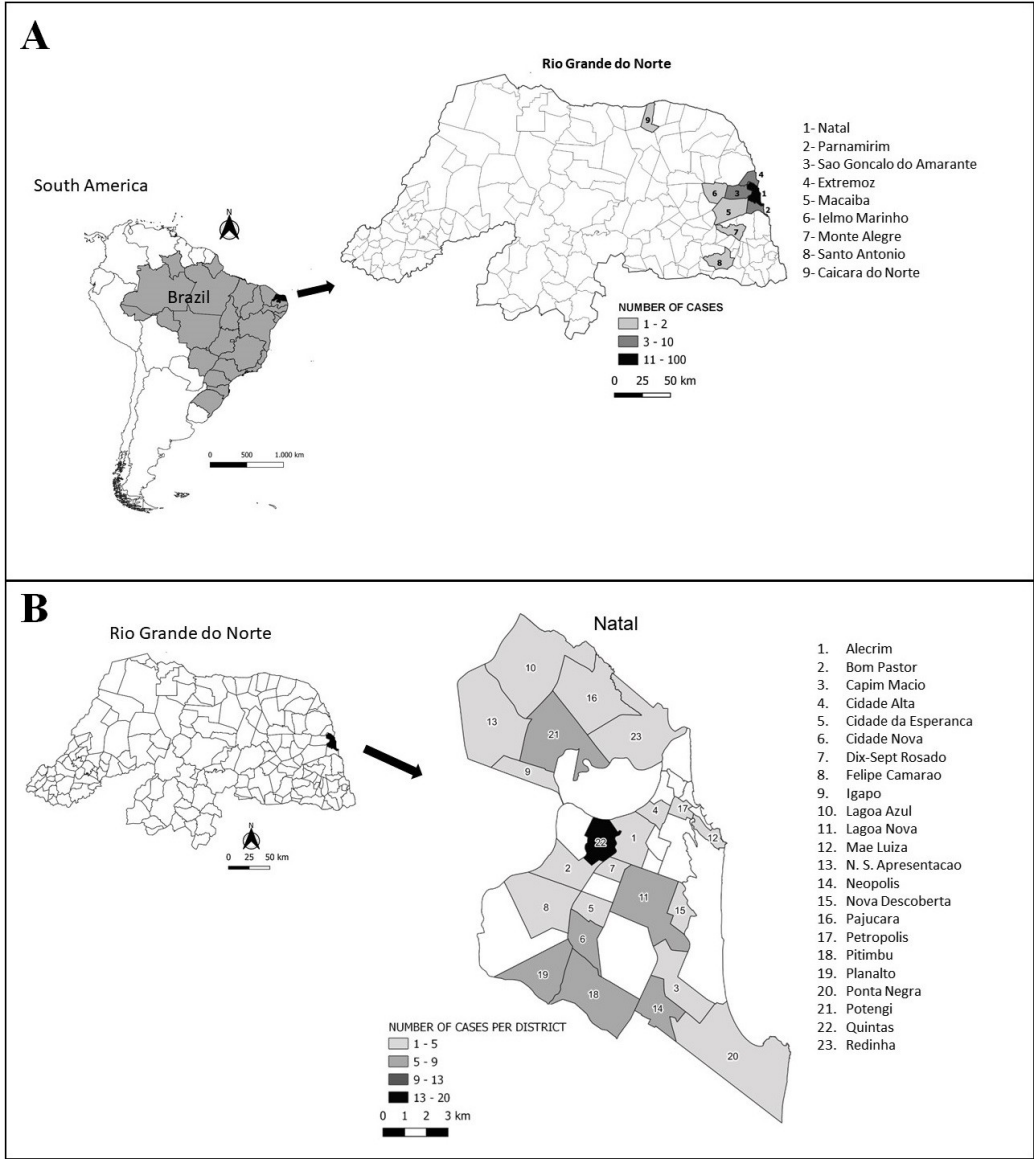
Spatial distribution of cat-transmitted sporotrichosis in Rio Grande do Norte state, Northeast Brazil from October 2016 to December 2019 (A). The most affected area is Quintas district, in the West Zone of the metropolitan region of Natal (B).

All the clinical cases occurred in urban areas, 98 (80.3%) patients lived in the city of Natal, Rio Grande do Norte state, Brazil. Among these, 45 cases (45.9%) occurred in the West Zone, with the highest concentration in the Quintas district (17 cases), followed by 17 cases in the South Zone (27.6%), 20 cases in the North Zone (20.4%) and six in the East Zone (6.1%). In addition, 23 cases (18.8%) occurred in neighboring municipalities (metropolitan region of Natal; Fig 2A and 2B). The address information is missing for a single patient.

### Phenotypic and molecular identification

Of the 122 cases, 71 (58.2%) of them were only clinically diagnosed (no mycological examination and PCR were performed), while 51 (41.8%) patients had a mycological examination, with the recovery of the etiological agent in culture and further PCR confirmation of the species involved. The phenotypic analysis, through the observation of macro and micromorphological characteristics, allowed the identification of the isolates at the genus level, being all initially identified as *Sporothrix* sp. For accurate identification at the species level, all isolates were subjected to molecular analysis. After PCR reaction and subsequent electrophoresis, a 469 bp DNA fragment product was observed using primers Sbra-F and Sbra-R, leading to the identification of 50 strains as *S*. *brasiliensis*, whereas a single sample showed no PCR amplification (patient 114, who reported barbed-wire injury).

### Treatment

One hundred and eighteen patients (96.7%) received an oral itraconazole treatment regimen, with daily doses of 200 mg; three of them were treated with saturated potassium iodide solution using 10 oral drops daily, and patient 77 presented spontaneous regression, using itraconazole for only 7 days. The treatment performed with itraconazole had an average duration of 12 ± 3.5 weeks, mode and median of 13 weeks, ranging from four to 25 weeks. The treatment carried out with potassium iodide was used for three patients, with an average of 9 ± 3 weeks ranging from 7 to 13 weeks. However, the length of treatment for 9 patients was missed during the study (Table 1).

The length of treatment was not influenced by the patient´s age or sex (*P*˃0.05). However, the most prolonged period of treatment (25 weeks) was necessary for a male patient aged 58 years old (56), and the shortest treatment length (4 weeks) was presented by four patients, 2 males aged 34 (patients 78 and 87) and another one who was 37 years old (patient 82), and a female patient aged 17 years old (patient 59). Nevertheless, when we have analyzed the length of treatment with itraconazole for each clinical form separately, it was possible to observe a statistically significant shorter period of time needed to treat patients with the fixed cutaneous form (duration of 11 ± 3.3 weeks, mode of 11.5 and median of 13 weeks, ranging from 4 to 19 weeks) when compared to those with lymphocutaneous lesions (duration of 12 ± 3. weeks, mode and median of 13 weeks, ranging from 1 to 25 weeks; *P* = 0.03).

In addition, 5 patients needed dose adjustment for itraconazole as follows: patient 38 who needed an increase in itraconazole dose from 200 mg/daily to 400 mg/daily after 7 weeks of treatment; patients 111 and 114 who started using 300 mg/daily after 2 weeks of treatment; and patients 101 and 108 who had their doses adjusted to 300 mg/daily of itraconazole after 5 and 4 weeks, respectively.

## Discussion

We describe here clinical case series of 122 cat-transmitted subcutaneous sporotrichosis mainly due to *S*. *brasiliensis* in Northeast Brazil. All the strains (except the strain obtained from patient 144) recovered from mycological cultures belong to this species, following the same trend of studies regarding zoonotic sporotrichosis performed in Brazil. It is important to note that *S*. *brasiliensis* was restricted to the South and Southeast of Brazil until mid-2010 [23]. Other *Sporothrix* spp., mainly *S*. *schenckii sensu stricto*, have been less related to transmission by cats in a few countries, including the United States, India, Malaysia, Argentina, Mexico, and Panama [17]. Another molecular technique with a better discriminatory power (such as rDNA sequencing) is mandatory to identify the strain obtained from patient 144 accurately. He reported injury when trying to go through a barbed-wire fence. We cannot rule out the possibility that *S*. *chilensis* [24] is the etiological agent of this case because this species has been recently described in 4 cases of sporotrichosis in the same Brazilian region (Northeast) of the present study [18].

Pubmed searches using the terms “*Sporothrix brasiliensis*” and “sporotrichosis cases” only retrieved 42 results. It is worth mentioning that most of these publications are rather related to feline sporotrichosis itself or even to basic sciences investigations regarding *S*. *brasiliensis* virulence. To the best of our knowledge, this is the first case series with a high number of patients seen in an area spanning nearly 2,700 km of distance from the epicenter in Rio de Janeiro, showing the spreading of the epidemic in other parts of Brazil (Northeast region).

Gremião *et al*. [25] reported the expansion of cat-transmitted sporotrichosis in Brazil. Rio de Janeiro researchers have been publishing high frequent emergent human cases of zoonotic transmission sporotrichosis since the late nineties [26], with almost 5,000 cases recorded from 1997 to 2011 [27]. However, since 1998, Brazil is under the geographic expansion of zoonotic sporotrichosis toward different regions of this continental-size country [26].

Cases of human sporotrichosis have been reported in almost all the 26 Brazilian states, but those directly related to cat transmission due to *S*. *brasiliensis* are highly concentered within Southwest and South regions [8], such as Sao Paulo [28], Espirito Santo [29] and Minas Gerais [30]. Recently, a case series of human zoonotic sporotrichosis has been described in Brasilia (Federal District; Midwestern Region) [31]. Only three other publications from the same region of the present study were found, two of them from Pernambuco state [18,32] and a case report of primary pulmonary sporotrichosis in an apparently immunocompetent female patient published by our group [7]. Apart from Brazil, cat-transmitted sporotrichosis was only reported recently in the North area of Buenos Aires province, Argentina, specifically in rural areas. Small outbreaks have been reported involving felines, cat owners, and even a veterinary doctor who examined a sick cat [33], proving that the spreading of the epidemic is already happening in South America. Since then, 21 cases of human sporotrichosis and 24 of feline sporotrichosis have been reported in the literature, reaching unprecedented levels, with a four-fold increase of cases of *S*. *brasiliensis* infections in humans from 2011 to 2019 [34].

Female patients were the vast majority of individuals found in our study. This finding is thought to be related to the high number of women who work as housewives or cleaners in Brazil and are in closest contact with animals at home. Similar results were obtained by other authors who investigated cat-transmitted sporotrichosis [35,36].

The upper limb was the most frequent body site found in the present study, followed by the lower limb and face. It is worth mentioning that these body sites are the most frequently affected, according to different studies [11]. Face lesions are described as more often observed in children and adolescents [37], corroborating with our results. Lymphocutaneous and fixed cutaneous clinical forms were found in our study. In cutaneous forms, the infection happens after injury with epidermis disruption. After fungal conversion into the host body, yeast cells may remain localized in host tissues (fixed forms) or disseminate to adjacent lymphatic vessels in the case of lymphocutaneous form. More rarely, where there is hematogenous dissemination, the cutaneous-disseminated form is found [11].

The fact that 39.4% of our patients had the fixed form is an unusual finding. Some authors believe that continuous exposure to small amounts of conidia could gradually confer immunity, specifically in patients with previous *Sporothrix* spp. traumatic inoculation preventing dissemination through the lymphatic system [38]. Nevertheless, in a study conducted in Iran, where sporotrichosis is a rare clinical condition, 7 out of 9 patients had the fixed cutaneous clinical form. These results suggest that this clinical form may be predominant in some regions of the globe [39].

Erythema nodosum in patients with cat-transmitted sporotrichosis was firstly reported by Galhardo *et al*., 2002 [40]. It has been considered a clinical syndrome possibly related to a hypersensitivity reaction due to inflammatory infection, neoplasm, and medication’s adverse effect. It is an acute eruption of erythematous nodules in lower limbs that may be followed by fever, malaise, and arthralgia. Erythema multiforme has also been previously described in zoonotic sporotrichosis. It is recognized as a vesiculobullous disorder with variable manifestations in the skin and is also considered a hypersensitivity disease [41].

Interestingly, we also had a 45-years-old female patient who used to treat a sick cat diagnosed with sporotrichosis. She denied bits and scratches and reported pain in her itchy and watery right eye which started 8 days after the appearance of subcutaneous sporotrichoid lesions in her left forearm. Schubach *et al*., 2005 [42] reported two cases of female patients presenting conjunctival granulomatous lesions with hyperemia, secretion, and edema. The authors reinforce the fact that conjunctival lesions are rarely observed in patients with sporotrichosis. In a study with 566 patients with sporotrichosis, only 13 (2.3% of them) had conjunctival lesions [42]. Because ocular manifestations of sporotrichosis are usually caused either by traumatic injury or due to hematogenous dissemination [43], only very few cases of primary granulomatous conjunctivitis have been reported in the literature [36,44,45].

Another interesting finding is that more than 36% of patients used to live in Natal city West Zone. This is also the region of the city with the highest number of cases with feline sporotrichosis. In fact, the veterinary doctors claim that this is the epicenter of feline and human sporotrichosis in Natal city. The first human and feline cases found at the beginning of the outbreak were described there. We hypothesize that this finding is due to the fact that this is one of the lowest income regions of the city, where cat owners usually do not have economic conditions to treat their animals. In addition, houses are divided by the same walls, which may favor feral and domestic cats contact and fights.

The fact that *S*. *brasiliensis* cells are already in the yeast form even colonizing cat´s claws and oral cavities may somehow favor the human infection because the fungus is well adapted to a warmblood animal, instead of the usual filamentous forms found in the environment, where temperatures are usually bellow 30°C. Therefore, conversion to the parasitic phase is not necessary when it reaches the human host [46,47]. In addition, it has been shown that *S*. *brasiliensis* can form biofilms on cat claws, which can lead to greater fungus persistence and better transmission during traumatic inoculation [48]. In addition, quite a few investigations reported *S*. *brasiliensis* as more virulent than *S*. *schenckii sensu stricto*. This fact may have influenced the spreading of cat-transmitted sporotrichosis in our continental-size country [49–51].

Finally, our patients were successfully treated with itraconazole, and therapeutic cure was observed in 100% of them. The most common antifungal drug to treat the patients was itraconazole. This is considered the drug of choice to treat subcutaneous sporotrichosis [52]. Interestingly, the treatment performed with this antifungal drug had a median duration of 13 weeks, 3 weeks before than what was observed by Almeida-Paes *et al*. [36]. This finding may be related to the doses used in the present study (200 mg/daily) instead of 100 mg/daily reported by these authors. Important to note dose adjustments and spontaneous regressions of sporotrichosis have also been observed in the present study.

Our study reported 122 cases of subcutaneous sporotrichosis in Northeast Brazil. One hundred and fifteen of them reported cat transmission. Diagnosis PCR revealed that 71 of them were due to *S*. *brasiliensis*. The majority of patients were female (70.5%). Patient’s age ranged from five to 87 years old. Most patients had the lymphocutaneous form (58.2%), but a relatively high percentage was found for the fixed cutaneous form (39.4%). Patients were successfully treated with itraconazole and potassium iodide. A limitation of our study is that in quite a few occasions, the diagnosis of zoonotic sporotrichosis was only clinical and not based on mycological examination and further identification with molecular biology methods. We are currently working on virulence factors expression and antifungal susceptibility testing of these isolates. Our findings are very similar to other publications of different Brazilian states reinforcing the idea of an epidemic and outbreak of feline sporotrichosis are underway in Brazil and Argentina, highlighting the spreading of the Disease toward South America.
